# HIV Reservoirs Across Multiple Tissues: From Heterogeneous Mechanisms to Therapeutic Targeting

**DOI:** 10.3390/microorganisms14040844

**Published:** 2026-04-09

**Authors:** Ya-Lan Wu, Gong-Wang Lan, Lin-Ying Jiang, Xin Chen

**Affiliations:** School of Basic Medical Sciences, Gannan Medical University, Ganzhou 341000, China; wuyalan1996@163.com (Y.-L.W.); 15297813449@163.com (G.-W.L.); 15880109631@163.com (L.-Y.J.)

**Keywords:** HIV reservoir, tissue heterogeneity, immune microenvironment, viral latency, anatomical sanctuary, drug penetration, targeted therapy

## Abstract

Despite effective antiretroviral therapy, HIV persists in diverse tissue reservoirs that pose major barriers to a cure. This review examines the heterogeneous maintenance mechanisms of HIV reservoirs in lymph nodes, intestinal mucosa, and the central nervous system (CNS). It analyzes how distinct tissue microenvironments—including immune-privileged niches, specialized cellular subsets, and local signaling networks—govern viral persistence and latency. Lymph nodes function as a dynamic hub interconnected with systemic reservoirs; the intestinal mucosa represents a site shaped by barrier integrity, microbial translocation, and mucosal immunity; the CNS constitutes a compartmentalized sanctuary protected by the blood–brain barrier. The review further discusses tissue-specific antiretroviral drug penetration and targeted clearance strategies, providing a foundation for developing multi-site intervention approaches toward HIV cure.

## 1. Introduction

Since the first report of acquired immunodeficiency syndrome (AIDS) in 1981 [1], over four decades of research have been dedicated to achieving a clinical cure for human immunodeficiency virus (HIV). Although antiretroviral therapy (ART) has significantly reduced AIDS-related mortality and curbed the global pandemic [2], HIV infection remains incurable. The fundamental obstacle is the virus’s ability to integrate into the host cell genome as provirus and establish viral reservoirs with long-term survival and self-renewing capacity [3,4].

The concept of the reservoir encompasses two interconnected dimensions: At the cellular level, the reservoir primarily resides within memory CD4^+^ T cell subsets possessing long-term survival potential [5], also including follicular helper CD4^+^ T cells (Tfh) [6], macrophages [7], and microglial cells within the central nervous system (CNS) [8]. These cells can maintain HIV proviral latency under ART pressure through mechanisms such as epigenetic silencing and mediating HIV immune evasion. At the anatomical level, over 98% of viral RNA-positive cells are actually located in lymphoid tissues, with blood accounting for only a minimal fraction [9].

Crucially, viral reservoirs exhibit significant heterogeneity across different tissues [10,11,12]. This heterogeneity manifests not only as differences in the number of infected cells but also as variations in maintenance mechanisms: How do the immune microenvironment, cellular composition, and signaling networks of different tissues influence the persistence of the viral reservoirs? Addressing this question constitutes a key scientific challenge for achieving an HIV cure. Although research models based on peripheral blood mononuclear cells (PBMCs) have yielded important progress [5,13], they struggle to fully replicate the complex cellular interactions and immune regulation within the tissue microenvironment, significantly hindering the clinical translation of related therapeutic strategies.

In this context, this review focuses on three major HIV reservoir tissues—lymph nodes, intestinal mucosa, and the CNS—to systematically elaborate how their distinct immune microenvironments shape the heterogeneous maintenance of viral reservoirs. Building on this foundation, we analyze the heterogeneous penetration of ART drugs within these three tissues and review targeted clearance strategies aimed at tissue-specific reservoirs. By integrating research progress across the three levels of “maintenance mechanism–drug penetration–clearance strategy,” this review aims to provide a theoretical basis for developing HIV reservoir research models that more closely reflect physiological conditions and offer new perspectives for advancing tissue-targeted reservoir intervention strategies.

## 2. Molecular Definition and Tissue Localization of the HIV Reservoir

Proviruses are not static or homogeneous entities. At the molecular level, the proviral pool comprises both intact and defective proviruses; at the anatomical level, their distribution exhibits significant tissue preferences. Precisely dissecting heterogeneity across these two dimensions is the logical starting point and core prerequisite for understanding reservoir maintenance mechanisms and designing targeted clearance strategies.

### 2.1. Intact Versus Defective Proviruses

The HIV proviral reservoir consists of two distinct molecular entities: Intact proviruses, possessing a complete genome and full replicative potential, are the primary sources of viral rebound upon ART interruption, conforming to the strict definition of a viral reservoir. Defective proviruses, containing large deletions or lethal hypermutations mediated by factors like APOBEC3G, cannot produce replication-competent progeny [14]. Although lacking replicative capacity, their transcriptional products can still mediate immunopathological damage, particularly within specific tissue microenvironments like the CNS.

High-throughput sequencing studies have revealed that defective proviruses dominate numerically in individuals on suppressive ART. Analysis using near-full-length individual viral genome sequencing showed that up to 97.6% of proviruses harbored lethal defects, with only 2.4% classifiable as intact [15]. Furthermore, studies using the intact proviral DNA assay (IPDA), which allows large-scale quantitative detection, confirmed that among one million CD4^+^ T cells, the total number of proviruses is approximately 755, with intact proviruses numbering around 54 [16]. After accounting for assay-related technical biases, the proportion of intact proviruses was estimated to be approximately 8% of the total proviral population, corresponding to a defective-to-intact provirus ratio of approximately 12.5:1 [16].

Intact and defective proviruses not only differ vastly in static abundance but also exhibit distinct characteristics in their in vivo dynamics and ex vivo proliferative capacity. During long-term ART suppression, the intact proviral reservoir shows a slow exponential decay (half-life of about 44 months) [15,17], whereas the dynamics of defective proviruses are more complex and variable. This difference is particularly pronounced in ex vivo clonal expansion assays: cells harboring defective proviruses can undergo up to 1000-fold clonal expansion upon T-cell receptor (TCR) stimulation, while cells carrying intact proviruses are rarely detected and proliferate poorly [15]. This suggests that viral integrity itself may impose constraints on host cell proliferative capacity; reactivation of intact virus likely induces cytopathic effects or immune clearance, favoring a quiescent state for cells harboring intact virus.

### 2.2. Precise Quantification Technologies for Proviruses

Accurately dissecting the distinct features of intact versus defective proviruses relies on advances in detection technologies. Traditional methods have inherent limitations. The quantitative viral outgrowth assay measures replication-competent virus by activating latent virus ex vivo, but single-round stimulation fails to induce all latent viruses, leading to underestimation of the true reservoir (i.e., intact proviruses) [14,15]. Conversely, widely used polymerase chain reaction (PCR), while sensitive, cannot distinguish intact from defective proviruses, and its results are confounded and inflated by the abundant defective proviruses, leading to overestimation of the reservoir [14].

To overcome this challenge, the IPDA technology was developed. This assay employs droplet digital PCR to simultaneously detect two critical regions of the viral genome at the single-molecule level, enabling effective discrimination and direct quantification of intact versus defective proviruses [15], providing a tool that more accurately reflects the true size of the HIV reservoir.

However, molecular quantification of proviruses represents only half the picture. Equally critical is elucidating the spatial distribution and cellular localization of the virus within tissues. While traditional tissue homogenate PCR can detect total viral nucleic acids, it cannot distinguish whether the signal originates from many cells carrying few viral copies or few cells carrying many copies [9]. Recently developed techniques like RNAscope and DNAscope in situ hybridization allow visualization of HIV RNA and DNA at single-cell resolution within intact tissue sections [9]. This technology not only pinpoints the specific cell types harboring the virus but also simultaneously assesses viral transcriptional activity, providing a powerful tool for understanding the true nature of the viral reservoir within the tissue microenvironment.

It is important to note that while technologies like IPDA can distinguish intact from defective proviruses, much research on proviral dynamics, host interactions, and tissue distribution still uses total HIV DNA as the observational target [5,11,13], constrained by the extremely low abundance of intact proviruses in vivo and sampling difficulties. Furthermore, the unique role of defective proviruses in mediating immunopathology makes them an indispensable component for understanding the full reservoir landscape. Therefore, in the subsequent discussion of this review, unless otherwise specified, “reservoir” refers broadly to the total HIV DNA within the body, aiming to integrate existing evidence from a broader perspective and subsequently explore the heterogeneity of viral distribution between blood and tissues.

### 2.3. Heterogeneity of Blood and Tissue Reservoirs

Peripheral blood is often considered a critical window for observing tissue viral reservoirs. Given that CD4^+^ T cells reside in both tissues and circulating blood, blood samples offer advantages in accessibility and repeatable sampling, whereas viral reservoirs in solid tissues are difficult to sample directly and monitor dynamically. But do viral reservoirs fundamentally differ between blood and tissues? In fact, peripheral blood circulation primarily serves as a conduit for migration and recirculation of immune cells like CD4^+^ T cells, not as the primary site for reservoir enrichment; the main “immune hubs” are located in lymphoid tissues, while mucosal tissues like the gut constitute key sites for immune responses and viral seeding [10]. Thus, peripheral blood reveals only the tip of the iceberg regarding the body’s tissue reservoirs and cannot fully or accurately reflect the true scale and characteristics of viral reservoirs at the tissue level.

Dominance of Lymphoid Tissues: Over 98% of viral RNA-positive cells are distributed in lymphoid tissues (lymph nodes, gut, spleen, etc.) [9]. Although the proportion of HIV DNA-carrying memory CD4^+^ T cells is similar between lymph nodes and peripheral blood, the absolute size of the viral reservoir in lymph nodes is substantially larger, owing to their much higher cellular density and total CD4^+^ T cell count [5,18]. However, using a Tat mRNA‑containing lipid nanoparticle in combination with panobinostat as a latency reversal strategy, detection of p24^+^ cells was found to be more probable in blood than in lymph nodes [11]. Concurrently, IPDA detection showed slightly higher abundance of intact proviruses in blood compared to lymph nodes [11]. These findings suggest that while lymphoid tissues harbor the largest total reservoir, their latency reversal efficiency might be lower than in blood.

Cellular Heterogeneity: In blood, central memory CD4^+^ T cells (Tcm) and transitional memory CD4^+^ T cells (Ttm) are the primary carriers of HIV provirus, with effector memory CD4^+^ T cells (Tem) also contributing [5,11]. Similarly, in lymph nodes, the reservoir primarily consists of Tcm and Ttm, including tissue-resident memory CD4^+^ T cells (Trm), whereas Tem are rare [11,12]. Notably, cytotoxic CD4^+^ T cells expressing granzyme A, induced by chronic inflammation, are also reservoir cells but are scarce in lymph nodes and found predominantly in blood [11].

Molecular Phenotypic Heterogeneity: Reservoir cells display significant tissue-specific differences in molecular phenotypes. In peripheral blood, memory CD4^+^ T cells carrying intact provirus predominantly exhibit an effector memory phenotype, significantly upregulating various immune checkpoint molecules—such as programmed cell death protein 1 (PD-1) and T cell immunoreceptor with Ig and ITIM domains (TIGIT)—as well as their corresponding ligands, including herpesvirus entry mediator and programmed death-ligand 1. Their key feature is the ability to resist killing by cytotoxic T lymphocytes and natural killer (NK) cells and to suppress viral transcription, thereby evading host immune recognition [12]. In contrast, intact provirus reservoir cells in lymph nodes, while also partially expressing some of these immune checkpoint molecules, are more significantly enriched in Tfh and Trm phenotypes, characterized by high expression of CD44, CD28, CD127 (interleukin-7 receptor), and interleukin-21 receptor, suggesting their core features are more aligned with enhanced cell survival and anti-apoptotic capacity [12]. Furthermore, in studies using total HIV DNA, reservoir cells across different tissues commonly show high expression of the anti-apoptotic molecule B-cell lymphoma 2 [11]. PD-1, TIGIT expression, and immune activation levels in lymph node Tcm and Ttm are significantly higher than in peripheral blood, a difference particularly pronounced in individuals with poor CD4^+^ T cell recovery [18]. Although these results differ somewhat from the phenotypic features described above for intact proviruses, it should be noted that this study did not distinguish between intact and defective genomes, potentially influenced by populations like Tfh highly expressing C-C chemokine receptor type 5 (CCR5) and PD-1 in lymph nodes. Nevertheless, this observation indirectly confirms that the overall HIV DNA burden is indeed higher in lymph nodes than in blood.

Differences in Maintenance Mechanisms: In patients on effective suppressive ART, different mechanisms drive the maintenance of these cellular subsets. In blood, long-term reservoir maintenance primarily relies on homeostatic proliferation of Ttm mediated by cytokines like interleukin 7 (IL-7), with chronic clonal expansion of Tcm playing an auxiliary role [5]. In contrast, TCR stimulation driven by antigen recognition can trigger robust clonal expansion but contributes relatively less to sustaining the overall reservoir size [5]. This dichotomy between blood and tissue is further exemplified by the distinct properties of α4β7^+^ CD4^+^ T cells. In the circulation, these cells are uniquely enriched for proliferative transcriptional programs [19]. However, once they seed tissues like the gut, their counterparts in the rectal mucosa downregulate this program and instead exhibit a core tissue-residency gene expression signature driven by the local microenvironment [19]. This observation underscores that tissue reservoir maintenance is dominated by tissue microenvironment-specific regulation: involving not only the synergistic action of local cytokines like IL-7 but, more critically, depending on tissue-unique microenvironments and factors—such as hypoxic conditions in lymph nodes shaping subsets like CD73^+^CD4^+^ T cells [20], the role of retinoic acid (RA) and the mucosal addressin cell adhesion molecule-1 (MAdCAM-1) on endothelial cells in gut tissue [21], and the unique immune milieu of the CNS coupled with a vicious cycle of chronic inflammation triggered by viral proteins [22,23]. These tissue-specific factors collectively sustain the latent tissue reservoir.

**Figure 1:**
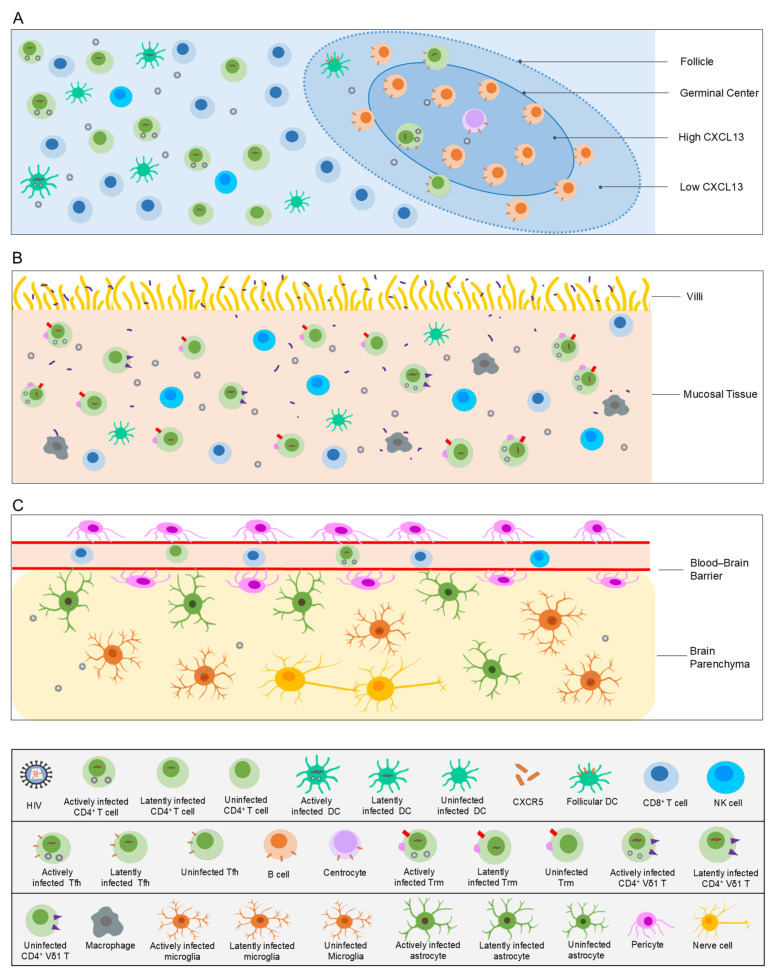
Overview of major HIV reservoirs in three anatomical compartments. (**A**) Lymph nodes. Within the germinal center of lymphoid follicles, high expression of C-X-C motif chemokine ligand 13 establishes a unique immune-privileged microenvironment. CD8^+^ T cells, which typically do not express CXCR5, are unable to enter the follicle, rendering follicular helper CD4^+^ T cells the core reservoir at this site. (**B**) Intestinal mucosa. The complex microbial environment and the relatively loose mucosal structure influence local immune reconstitution. Tissue-resident memory CD4^+^ T cells are identified as the principal viral reservoir in this compartment. (**C**) Central nervous system (CNS). The blood–brain barrier restricts the penetration of drugs and immune cells. Microglia within the brain parenchyma serve as the primary long-lived latent reservoir.

Peripheral blood serves as a “window” into tissue reservoir, offering a crucial entry point for understanding HIV latency. However, fundamental differences exist between blood and tissues regarding reservoir size, cellular composition, molecular phenotypes, and maintenance mechanisms. Blood cannot capture how tissue-specific microenvironments confer stubborn persistence upon the reservoir. To genuinely achieve a “cure,” the research perspective must extend from blood to the lymph nodes, gut mucosa, and CNS—the primary body of the reservoir “iceberg” (Figure 1)—and delve into how the microenvironments of these core regions shape and sustain latent reservoirs, thereby providing the critical theoretical foundation for targeted clearance strategies.

## 3. Lymph Nodes: The Hub Reservoir and Its Complex Microenvironmental Regulation

Lymph nodes are the central sites for adaptive immune responses and serve as the primary and most complex anatomical hubs for HIV persistence [9]. Their unique architecture, dynamic cellular networks, and specialized immune microenvironment collectively create a niche that supports potent antiviral immunity while paradoxically providing long-term sanctuary for the virus, posing a major challenge for eradication.

### 3.1. Follicular Architecture and the Immune-Privileged Sanctuary

The compartmentalization of lymph nodes dictates the spatial distribution of immune cells and viruses. The paracortex is the primary site for T cell activation, while the lymphoid follicles in the superficial cortex are where B cell maturation and germinal center formation occur. Germinal centers are enriched with Tfh [5], a highly activated subset critical for B cell help and a key reservoir for both HIV replication and latency [13]. This microenvironment is characterized by high expression of the chemokine C-X-C motif chemokine ligand 13 (CXCL13), recruiting C-X-C motif chemokine receptor 5 (CXCR5)-expressing B cells and Tfh into the follicles [24]. In contrast, potent cytotoxic CD8^+^ T cells, which typically lack CXCR5, are largely excluded from these areas [25]. This mechanism, evolved to optimize humoral immunity, is co-opted by HIV to establish a functional “immune-privileged” sanctuary within the follicles, representing a core obstacle to latent reservoir clearance.

### 3.2. Dynamic Nature and Systemic Interconnectedness of the Reservoir

The viral reservoir within lymph nodes is neither static nor isolated. Phylogenetic studies have demonstrated that clonally expanded infected cells, harboring identical proviral integration sites, are widely disseminated across multiple anatomical compartments—including blood, lymph nodes, and the gut mucosa—and actively migrate between these sites even during ART suppression [11]. Early in infection, viral populations in blood and lymph nodes are already highly admixed, indicating the continuous systemic trafficking of HIV-infected CD4^+^ T cells [26]. Post-mortem analyses further confirm that expanded clones carrying intact proviruses are shared across distinct tissues, underscoring that clonal proliferation and migration are system-wide phenomena [27]. Therefore, the lymph node reservoir should be viewed as a dynamic hub intricately interconnected with the systemic viral pool. This interconnectedness implies that any effective eradication strategy must simultaneously target local sanctuary microenvironments and adopt a systemic intervention perspective.

### 3.3. Roles of Key Cellular Subsets

Tfh: Tfh constitute the largest component of the viral reservoir in lymph nodes and harbor significantly higher levels of HIV RNA compared to other reservoir cells in blood or lymph nodes [13,26]. Although their contribution to the viral replication pool is minimal during acute infection, Tfh populations expand during the chronic phase, becoming major sites for both active viral replication and the establishment of latency, thereby playing a decisive role in maintaining the viral set point [26]. During ART-suppressed infection, PD-1^+^ Tfh within lymph nodes constitute a primary reservoir for ongoing HIV-1 transcription [28]. The size of the viral reservoir correlates with time on ART. Long-term treatment leads to germinal center involution and a concomitant decline in both the Tfh population and its associated reservoir. Notably, however, the HIV reservoir within lymph node PD-1^+^ Tfh is more stable than that in other memory CD4^+^ T cell subsets, persisting for at least 12 years [28].

Dendritic cells (DCs): Recent studies indicate that lymph node DCs constitute an underestimated reservoir for HIV and are capable of inducing viral replication [29]. Under conditions of viral suppression, viral sequences induced from DCs and Tfh show high homology and an absence of drug resistance mutations. Notably, HIV RNA levels in migratory DC subsets are comparable to those in Tfh [29]. As the reservoir decays with prolonged ART, resident DC subsets exhibit the slowest decline in viral DNA levels compared to Tfh and migratory DCs. This suggests that resident DCs may represent an exceptionally stable reservoir compartment, potentially exceeding the stability of the Tfh reservoir [29].

Specialized CD8^+^ T Cells: During chronic infection, a subset of HIV and simian immunodeficiency virus (SIV) specific CD8^+^ T cells can upregulate CXCR5 and differentiate into follicular cytotoxic T cells (Tfc), which thereby gain access to germinal centers and breach the typical spatial segregation [30]. Although Tfc are present in limited numbers within follicles and often exhibit an exhausted phenotype (e.g., high PD-1 expression), they retain cytotoxic potential via the granzyme-perforin pathway [31]. SIV studies indicate that Tfc can suppress viral replication in Tfh reservoirs under ART and display features of immunosenescence [32]. Furthermore, research has identified a CXCR5^+^CD8^+^ T cell population exhibiting stem-like properties (TOX^hi^TCF1^+^CD39^+^) [33]. We propose that this population represents precursors to Tfc, as it has been shown to differentiate into terminally differentiated CD8^+^ T effector cells. Although these precursor cells themselves bear an exhausted phenotype, they retain effector function, albeit with reduced cytotoxicity. Residing both inside and outside follicles, they contribute to viral suppression within the lymph node reservoir [33]. Collectively, these specialized CD8^+^ T cell subsets represent promising targets for immunotherapeutic strategies.

CXCR5^+^ NK Cells: A recent study in a chronic simian-human immunodeficiency virus (SHIV) infection model has identified a subset of NK cells expressing CXCR5, demonstrating significant potential in mediating viral immune control [34]. By virtue of CXCR5 expression, these cells are able to accumulate within B cell follicles. Compared to conventional NK cells, intrafollicular CXCR5^+^ NK cells exhibit a highly activated phenotype and a distinct transcriptional profile, characterized by markedly enhanced effector functions and elevated expression of key cytotoxic mediators, including perforin, granzymes, and lysosomal-associated membrane protein 1 [34]. Furthermore, this subset co-expresses Fc fragment of IgG receptor IIa and Fc fragment of IgG receptor IIIa, conferring enhanced capacity for antibody-dependent cellular cytotoxicity (ADCC). These properties are closely linked to their in vivo antiviral activity: the abundance of CXCR5^+^ NK cells correlates negatively with both plasma viral load and the frequency of Tfh [34]. Mechanistic study further reveal that interleukin 12 (IL-12) and interleukin 15 (IL-15) signaling pathways are specifically upregulated in CXCR5^+^ NK cells, and in vitro experiments confirm that this cytokine combination effectively promotes the proliferation of CXCR5^+^ granzyme B^+^ NK cells, suggesting IL-12 and IL-15 are key microenvironmental factors driving the expansion and functional maintenance of this subset [34].

CXCR3^+^ Tfh: During HIV-1 infection, a subset of Tfh with a T helper 1-like phenotype, defined by C-X-C chemokine receptor type 3 (CXCR3) expression, plays a unique dual role. In acute infection, while the overall frequencies of Tfh and germinal center B cells remain stable, the proportion of proliferating CXCR3^+^ Tfh increases significantly [35]. The emergence of this activated cell population positively correlates with the generation of HIV-specific antibodies and functions such as ADCC and antibody-dependent cellular phagocytosis, highlighting its positive role in fostering effective humoral immune responses [35]. However, this subset also serves as a “Trojan horse” for viral infection: CXCR3^+^ Tfh are infected by HIV-1 at a significantly higher rate than their CXCR3^−^ counterparts, potentially rendering them important vectors for early viral dissemination within follicles [35].

Other Key Lymph Node Populations: Beyond the subsets described above, the lymph node microenvironment also shapes other critical cells with immunoregulatory and viral reservoir properties. In lymph nodes from HIV-infected individuals on combination ART, a population of FOXP3^hi^CD4^hi^ T cells is significantly enriched [36]. This subset exhibits features of regulatory T cells and possesses the capacity to suppress Tfh function, potentially contributing to the decline in Tfh numbers observed during ART [36]. Additionally, a unique population of CD3^+^CD20^+^ double-positive lymphocytes exists within lymph nodes. Their formation results from membrane exchange during frequent T cell–B cell contacts within the node, primarily involving T cells acquiring CD20, rendering them rare in peripheral blood [37]. This double-positive population includes both CD4^+^ and CD8^+^ T cells but predominantly consists of CD4^+^ Tfh. Importantly, these cells are susceptible to HIV/SIV infection, harboring SIV DNA at levels comparable to conventional CD4^+^ T cells, suggesting they constitute a lymph node-specific viral reservoir subset [37].

### 3.4. Immune Regulation and Viral Latency in the Lymphatic Microenvironment

The germinal centers of lymph nodes constitute a physiologically hypoxic microenvironment, resulting from sparse vasculature and high cellular metabolic activity. Hypoxia induces high expression of the ectonucleotidase CD73 on CD4^+^ T cells. CD73, in concert with CD39, catalyzes the hydrolysis of adenosine monophosphate to adenosine. Adenosine, via the A2A receptor signaling pathway, inhibits T cell immune activity and promotes HIV latency [20]. These CD73^+^CD4^+^ T cells display a dual phenotype of “deep latency yet high inducibility”. Specifically, they maintain profound viral latency by downregulating host factors involved in viral replication (e.g., GATA3, TOP2A), but exhibit high viral reactivation efficiency upon strong TCR stimulation (e.g., anti-CD3/CD28 treatment) [20]. Blocking CD73 or adenosine receptors in vitro can promote the reactivation of latent HIV, indicating that CD73^+^CD4^+^ T cells constitute a unique latent reservoir subset.

### 3.5. Central Role in Viral Rebound

The lymph node reservoir is a primary source of viral rebound upon ART interruption. Animal model studies confirm that plasma virus clones emerging early in rebound originate predominantly from lymphoid tissues, such as mesenteric lymph nodes, inguinal lymph nodes, and the spleen [38], with their contribution far exceeding that of virus from peripheral blood or intestinal mucosa [39]. This highlights the central role of lymph nodes as both a reservoir and a reactivation source in viral rebound.

Summary: As the linchpin of the viral reservoir, the persistence of HIV in lymph nodes is deeply rooted in the intricate interplay of three factors: anatomical structure, cellular composition, and microenvironmental signals. Structurally, germinal centers establish a physical “follicular immune sanctuary” via the CXCL13-CXCR5 axis, excluding the vast majority of cytotoxic CD8^+^ T cells and creating a natural haven for the HIV. Cellularly, a highly heterogeneous and dynamically complementary reservoir network is formed, with Tfh as the primary carrier, complemented by DCs, specialized CD8^+^ T cells and CXCR5^+^ NK cells possessing both migratory and killing potential, as well as FOXP3^hi^CD4^hi^ T cells and CD3^+^CD20^+^ double-positive lymphocytes shaped by the unique physiological environment. Microenvironmentally, hypoxia drives CD73^+^CD4^+^ T cells towards a phenotype of “profound latency yet high inducibility.” Critically, lymph nodes do not exist in isolation; infected cell clones continuously exchange with peripheral blood and the gut reservoir through systemic circulation, forming an interconnected network. This pivotal role establishes lymph nodes as the primary epicenter of viral rebound upon ART interruption. In summary, the lymph node reservoir represents a multi-layered complex, sheltered by immune-privileged structures, harbored by specialized cellular subsets, conditioned by the local microenvironment, and dynamically linked systemically.

## 4. Intestinal Mucosa: A Dynamic Barrier and Multifactorial-Maintained Viral Reservoir

The intestinal mucosa, endowed with a vast surface area, a dense immune cell network, and continuous microbe-host crosstalk, serves not only as a crucial barrier for host defense but also as a key site for extensive viral replication during acute HIV infection and persistent viral latency under long-term ART [40]. The viral reservoir here in is coordinately regulated by unique cellular subsets, the local microenvironment, and host genomic factors, forming a highly complex and dynamically maintained system.

### 4.1. Key Reservoir Cellular Subsets

Trm: The intestinal mucosa is enriched with Trm expressing CD69 and CD103, which are central to local immune surveillance and act as important HIV reservoir cells [10,41,42]. RA, a key factor in the intestinal microenvironment produced by DCs, synergizes with transforming growth factor-β (TGF-β) and MAdCAM-1 to drive the differentiation of naive CD4^+^ T cells into Trm-like cells expressing CCR5, α4β7, CD69, and CD103. This process significantly enhances their susceptibility to HIV, thereby facilitating the formation and replenishment of the intestinal reservoir [21].

CD4^+^ T Cells with Unique Exhaustion-Memory Phenotypes: In-depth studies have revealed that intact HIV proviruses in the intestine (particularly the colon) are not uniformly distributed but are highly enriched in a specific subset of mucosal CD4^+^ T cells: PD-1^+^TIGIT^−^CD27^+^ cells [40]. This phenotypic profile indicates that these cells have undergone antigen stimulation (PD-1^+^), exhibit certain exhaustion characteristics but have not entered a terminally exhausted state (TIGIT^−^), and retain proliferative capacity and long-term survival potential (CD27^+^) [40]. This unique cellular state creates a microenvironment conducive to viral latency and clonal maintenance, serving as an important cellular basis for the intestinal mucosal reservoir.

Dual Role of Vδ1 γδ T Cells: Vδ1 T Cells, a subset of γδ T cells in the intestinal mucosa, play a paradoxical dual role in HIV infection [43]. On one hand, a subset of Vδ1 T cells can be activated via T cell receptor mediation, upregulate CD4 expression, and subsequently become infected with HIV, contributing to the viral reservoir. Studies have shown that CD4^+^ Vδ1 T cells account for approximately 20% of all infected CD4^+^ T cells in the colon and small intestine, indicating that they represent a non-negligible viral reservoir in the intestinal mucosa. On the other hand, another subset of Vδ1 T cells possesses potent cytotoxic functions and can effectively suppress viral replication in infected cells [43]. Furthermore, similar to αβ CD8^+^ T cells, pretreatment of these Vδ1 T cells with the common γ-chain cytokine IL-15 can enhance their anti-HIV activity [43]. This “friend-or-foe” characteristic of Vδ1 T cells underscores the complexity and dynamism of the cellular composition of the intestinal viral reservoir.

Intestinal Epithelial Cells (IECs): IECs are widely distributed throughout the intestinal mucosa and, under physiological conditions, continuously interact with T cells, promoting HIV infection and the establishment of latent reservoirs in CD4^+^ T cells, particularly C-C chemokine receptor type 6^+^ T helper 17 cells [44]. As discussed previously, MAdCAM-1 expressed by IECs facilitates the differentiation of Trm-like cells, indirectly contributing to HIV infection and reservoir formation [21]. Beyond this, IECs can express major histocompatibility complex class II (MHC II) in response to interferon-γ and significantly enhance both productive and latent HIV infection in resting CD4^+^ helper T cells [44]. In activated CD4^+^ T cells, IECs not only augment HIV infection but also promote the establishment of latency. Notably, memory CD4^+^ T cells are more susceptible to IECs-mediated HIV infection compared to naive CD4^+^ T cells, a process dependent on the cytokine interleukin-6 but independent of the co-stimulatory molecule CD2 [44].

### 4.2. Specific Regulation by the Intestinal Microenvironment

Direct Regulation by Microenvironmental Factors: Gut-specific RA not only shapes target cells but also acts directly on already infected cells. By activating the mammalian target of rapamycin (mTOR) signaling pathway, RA promotes the replication of HIV-1 in macrophages [45]. This pathway exerts its promotional effects through two distinct mechanisms: first, it modulates the restriction factor sterile alpha motif and histidine-aspartate domain-containing protein 1 in macrophages, thereby attenuating the host cell’s intrinsic restriction on viral reverse transcription; second, it activates transcription that is dependent on both cyclin-dependent kinase 9 and RNA polymerase II, which enhances viral transcriptional efficiency and consequently facilitates the maintenance and renewal of viral reservoirs [45]. These findings suggest that targeting downstream pathways of RA, such as the mTOR pathway, may hold potential for therapeutic intervention.

Integrin α4β7 Promotes HIV Infection: α4β7, an intestinal homing receptor, is expressed on immune cells such as T cells and B cells. The natural ligand for α4β7 is MAdCAM-1 on IECs. This integrin can be directly bound by recombinant proteins and synthetic peptides derived from HIV/SIV gp120 [46]. Furthermore, similar to MAdCAM-1, the V2 loop of gp120 is capable of transducing signals through α4β7, leading to CD4^+^ T cell activation and proliferation [47]. This process facilitates HIV/SIV infection, thereby contributing to the expansion of the viral reservoir in intestinal mucosal. Notably, this binding and subsequent cellular activation can be effectively inhibited by soluble MAdCAM-1 and anti-α4β7 monoclonal antibodies, providing a rationale for targeting the intestinal homing pathway to intervene in the establishment and maintenance of the viral reservoir [46,47].

Vicious Cycle of Barrier Impairment and Inflammation: Immune activation following HIV infection leads to intestinal epithelial barrier damage, causing microbial translocation and driving systemic and local persistent immune activation. In turn, this chronic inflammatory microenvironment promotes the stabilization and maintenance of viral reservoirs, forming a vicious cycle of “viral persistence–immune activation and inflammation–barrier damage–microbial translocation–viral persistence” [48]. Studies have confirmed that the aberrant distribution of zonulin, a tight junction regulatory protein, is closely associated with intestinal barrier impairment and local inflammation (e.g., elevated interferon-α levels) [49], providing molecular evidence for this cycle. However, this vicious cycle is not insurmountable. During the early stage of immune compensation, the partial preservation of E-cadherin in the intestinal mucosa and the relative stability of γδ T cell function can, to a certain extent, constrain the inflammation-promoting vicious cycle driven by intestinal epithelial barrier impairment [50], although such compensatory effects ultimately become exhausted.

### 4.3. Potential Genomic-Level Influences

The impact of the chronic inflammatory microenvironment may extend to the host genomic level. Studies have demonstrated differential expression of endogenous retroviral elements (EREs) in colonic tissues of HIV-1-infected individuals, with the expression levels of specific elements (e.g., LTR19_12p13.31) showing a significant correlation with the frequencies of colonic immune cell subsets [51]. These observations suggest that aberrant EREs expression may reflect or mediate immune microenvironment dysregulation induced by HIV infection [51]. Furthermore, EREs may indirectly affect the establishment and maintenance of viral reservoirs through mechanisms such as epigenetics, thereby providing a novel molecular perspective for understanding the long-term persistence of viral reservoirs.

### 4.4. Reservoir Compartmentalization and Dynamics

In typical disease progressors, viral populations between the gut and peripheral blood are usually highly admixed, failing to form strict genotypic compartmentalization [52]. However, in specific populations (e.g., SIV-infected long-term non-progressors), gut tissues may harbor higher levels of residual virus and even exhibit significant local viral compartmentalization. This phenomenon correlates with differences in disease progression and immune control capacity [53]. Studies have indicated that medium- to long-term ART effectively reduces the size and diversity of gut viral reservoirs, even in elite controllers, highlighting the importance of sustained treatment [53]. These findings reveal marked heterogeneity in the dynamics of gut viral reservoirs, and the formation of strict compartmentalization depends on multiple factors, including infection stage, disease progression status, and treatment history.

Multiple studies have demonstrated that following effective ART for more than two years, the lymph node reservoir declines [9,28]. In contrast, within the intestinal mucosal tissue, the frequency of viral DNA-positive cells does not exhibit a significant decrease after ART [9]. This stark contrast underscores the particular resilience of the gut reservoir. The underlying mechanism is likely attributable to the unique microenvironment of the intestinal tissue: persistent low-level inflammation driven by ongoing microbial translocation, coupled with abundant homeostatic proliferation signals (e.g., IL-7), collectively sustain the stability of the gut viral reservoir.

Summary: The gut mucosal viral reservoir is not merely a passive latent viral pool, but a highly specialized system actively shaped by the tissue microenvironment, dynamically involving multiple cellular populations, and tightly coupled with the local pathological ecosystem. Its persistence stems from three core dimensions: First, the heterogeneity and plasticity of cellular carriers. Beyond Trm as the primary reservoir, a complex cellular network exists, including PD-1^+^TIGIT^−^CD27^+^ cells possessing both antigen-experienced and long-lived properties, Vδ1 T cells with dual functions in infection and killing, and even IECs themselves. Second, the direct drive and transformation by microenvironmental signals. Tissue-specific molecules such as RA, TGF-β, and MAdCAM-1 not only increase target cell susceptibility by shaping homing and differentiation but also directly regulate the latency and replication of already infected cells, converting physiological immune trafficking into pathological reservoir expansion. Third, the vicious cycle of barrier damage and chronic inflammation serves as the core driver. Persistent microbial translocation and low-grade inflammation provide an inflammatory niche conducive to viral persistence while continuously replenishing the reservoir pool through homeostatic proliferation signals, rendering it highly resilient even under ART. In summary, the gut reservoir is the product of long-term tripartite interactions among the “microenvironment, cells, and pathogen.”

## 5. Central Nervous System: An Immune-Privileged and Dynamically Compartmentalized Persistent Reservoir

The CNS, with its unique anatomical and immunological characteristics, constitutes a highly persistent and refractory reservoir for HIV, posing an exceptional challenge to eradication efforts. The blood–brain barrier (BBB), which is jointly formed by tight junctions between brain capillary endothelial cells, astrocyte end-feet, and pericytes, not only physically restricts the entry of immune effector molecules such as cytotoxic T cells and antibodies but also actively reduces the concentrations of many ART drugs in the brain parenchyma via efflux pumps (e.g., P-glycoprotein) highly expressed on endothelial cells [54,55]. This dual property of “immune privilege” and “drug sequestration,” combined with reservoir cells centered on microglia and specific T cell subsets, collectively sustains the long-term persistence of the CNS reservoir.

### 5.1. Major Reservoir Cells and Their Maintenance Mechanisms

Microglia: The Primary Resident Viral Reservoir. As resident immune cells of the CNS, microglia are widely recognized as the primary and most persistent HIV reservoir in the brain [8]. Autopsy studies have confirmed that intact proviruses can still be detected in microglia even after long-term ART [56]. Additionally, astrocytes and pericytes have also been identified as HIV reservoir cells, albeit with relatively low infection rates [57,58].

Migratory CD4^+^ T Cells: Key to Dynamic Replenishment and Reactivation. Although lymphocytes account for only approximately 1% of CNS cells compared to myeloid cells, their infection efficiency is 10 times higher [59]. Thus, migratory CD4^+^ T cells entering the CNS, due to their higher susceptibility to HIV, play a crucial role in the dynamic replenishment and reactivation of the viral reservoir. Studies have demonstrated that even during viral suppression, there are partially overlapping infected T cell clones between cerebrospinal fluid (CSF) and peripheral blood, indicating ongoing CD4^+^ T cell migration [60,61]. Following ART interruption, the rebounding viruses in the CSF are predominantly T cell-tropic (R5-tropic) clonally expanded viruses, suggesting that migratory CD4^+^ T cells are the primary contributors to CNS viral rebound [61].

Molecular Mechanisms of Maintenance and Reactivation: The persistence of the reservoir is intimately linked to the local microenvironment. For instance, astrocytes can capture extracellular Tat protein via endocytosis; accumulated Tat subsequently induces endosomal de-acidification, triggering strong pro-inflammatory signaling pathways such as nuclear factor-kappa B through Toll-like receptor (TLR) mediation. These pathways synergize with Tat to potently trans-activate latent proviruses [62]. Concurrently, the survival of latently infected pericytes depends on the activation of the AKT signaling pathway [63]. More importantly, even in the absence of intact provirus production, viral proteins (e.g., Tat, Nef) generated by reservoir cells exert neurotoxic effects [22,23], exacerbating local inflammation and thereby trans-activating latent virus. Furthermore, the maintenance of gap junction and hemichannel communication via Tat-mediated mechanisms can propagate inflammation [57], establishing a vicious cycle of “viral protein–inflammation–viral reactivation.”

### 5.2. Dynamic and Relatively Compartmentalized Viral Evolution

Compared with lymph nodes, the CNS viral reservoir exhibits more prominent genotypic compartmentalization. Although migration of infected CD4^+^ T cells occurs between the blood and the CNS, the vast majority of HIV-infected CD4^+^ T cell clones in the CSF are compartment-specific [60]. This compartmentalization becomes more pronounced during viral rebound: while viral loads in the blood and CSF are comparable during this period, with clonally expanded viral lineages emerging simultaneously, the proportion of clonally expanded lineages in the CSF is higher than that in the blood, and the dominant rebounding viral clonal lineages often differ [61]. Furthermore, viruses in the CSF frequently display unique phylogenetic characteristics and “amino acid signature patterns” [64]. Notably, this compartmentalization does not represent absolute isolation but involves dynamic bidirectional “seeding”; however, defective proviruses tend to accumulate within the CNS, forming a distinct viral population [65].

### 5.3. Early Establishment and Clearance Bottleneck of the Reservoir

The CNS viral reservoir is established very early during acute infection (even prior to seroconversion) and exhibits remarkable stability under long-term ART [66]. Studies have demonstrated that during viral suppression, the levels of intact and defective proviruses in brain tissue show no significant difference compared with the untreated phase [67,68], and the timing of ART initiation (within the chronic infection phase) does not appear to affect the final residual level of the CNS viral reservoir [69]. These findings highlight the recalcitrance of the CNS viral reservoir, as well as the fundamental limitations of current therapies in penetrating the BBB and eliminating infected brain cells.

### 5.4. The Unique Local Immune and Pathological Environment

Tempered Antiviral Response: To protect the delicate neural tissue, immune responses within the CNS are generally tempered. During acute infection, myeloid cells such as microglia undergo significant population restructuring (with altered gene expression also observed in bystander cells): the proportion of homeostatic subsets decreases, while specific activated subsets expand dramatically [59]. Microglia exhibit a prominent antiviral state, characterized by widespread upregulation of TLRs, substantial downregulation of MHC II molecules, general upregulation of major histocompatibility complex class I molecules and interferon signaling pathway-related genes, as well as significant upregulation of genes encoding type I interferon-inducible proteins. Additionally, microglia and macrophages are more prone to apoptosis during this phase [59]. This antiviral state may promote the apoptosis of infected cells and drive the virus toward deeper latency.

Antibody Compartmentalization: Compartmentalization of antibody function within the CNS also contributes to HIV latency. The neonatal Fc receptor on the BBB selectively recycles IgG antibodies with higher affinity from the CSF [70]. This selective permeability and recycling by the BBB result in a distinct antibody repertoire within the CNS compared to peripheral blood; antibodies in the CSF exhibit weaker functional coordination and binding capacity (e.g., ADCC) [70]. This functional antibody compartmentalization further impairs local immune clearance capacity. Even under ART, functional segregation of antibody effector molecule profiles still exists across different compartments [70].

Vicious Cycle of Neuroinflammation and Viral Replication: Persistent low-grade neuroinflammation in the CNS is a key driver of reservoir maintenance. HIV infection and associated neurotoxic proteins (e.g., Tat, Nef) can induce neuroinflammation [22,23], which in turn favors viral reactivation. More complexly, studies have demonstrated that pathological proteins associated with neurodegenerative diseases such as Alzheimer’s disease (e.g., α-synuclein fibrils) can significantly enhance HIV infection efficiency in both CD4^+^ T cells and microglia. Notably, the HIV envelope protein itself can form similar amyloid-like fibrils. These two types of fibrils may form a mutually catalytic positive feedback loop, exacerbating neural damage and viral persistence [71].

### 5.5. Host Genomic-Level Regulation

Viral integration and latency in CNS cells are also finely regulated by host genome architecture. Consistent with infected cells in peripheral blood, HIV-1 cDNA preferentially integrates into the intronic regions of transcriptionally active host genes in microglia, with enrichment near enhancers and boundaries of topologically associating domains defined by CCCTC-binding factor (CTCF) protein [72]. The chromatin accessibility of these regions facilitates viral integration and subsequent regulatory processes; studies have demonstrated that CTCF depletion significantly impairs the viral integration process [72]. Simultaneously, the host-encoded long non-coding RNA *NEAT1* can sequester viral genomic RNA in nuclear paraspeckles—preventing viral RNA nuclear export and the completion of viral assembly—thereby exerting an intrinsic antiviral effect [73]. This mechanism may influence the balance between viral replication and latency in the CNS.

Summary: The recalcitrance of the CNS viral reservoir stems from its multiple lines of defense: the physical and pharmacological shielding of the BBB, the resident cellular reservoir centered on microglia, the dynamic migration and high susceptibility of CD4^+^ T cell replenishment, the unique relatively compartmentalized viral evolution, and the inflammatory vicious cycle co-driven by viral proteins and host pathological proteins. These factors are intertwined, rendering the CNS one of the most formidable strongholds that must be conquered to achieve an HIV cure. Future strategies should focus on developing novel therapies capable of efficiently penetrating the BBB, specifically targeting brain reservoir cells, and modulating the local immune–inflammatory microenvironment.

## 6. Heterogeneity of ART Drug Penetration in Three Tissues

The ability of HIV to maintain low-level replication under ART stems not only from viral latency mechanisms but also from the limited and heterogeneous penetration of antiretroviral drugs into different tissues, constituting another critical factor in reservoir persistence. By comparing ART drug concentrations in peripheral blood, lymph nodes, ileum, and rectal tissue, Estes et al. found that, with the exception of certain drugs in the rectal mucosa, drug concentrations in nearly all tested tissues were lower than those in peripheral blood [9]. This systematically reveals the existence of a pharmacological tissue barrier.

### 6.1. Drug-Dependent Penetration and the Follicular Barrier in Lymph Nodes

As a primary reservoir site, drug penetration into lymph nodes is highly drug-dependent. First, the physicochemical properties of the drug itself determine its penetrative capacity. Studies have found that the active metabolites of nucleoside analogs (e.g., tenofovir, TFV; emtricitabine, FTC) exhibit the highest penetration in PBMCs and lymph nodes [74]. In contrast, the integrase inhibitor dolutegravir (DTG) and the protease inhibitor darunavir show moderate to low lymph node penetration [74], with DTG concentrations in lymph nodes reaching only 1.4% of those in blood, while the lymph node-to-plasma ratio for maraviroc is as high as 6.9 [75]. Second, the route of administration significantly affects drug distribution in specific lymph nodes (e.g., mesenteric lymph nodes); oral and subcutaneous administration enhance the accumulation of hydrophilic drugs (e.g., lamivudine) in lymph nodes compared to intravenous injection [76]. These findings suggest that therapeutic strategies targeting the lymph node reservoir must consider both drug molecular properties and administration routes to achieve effective coverage of infected cells within follicles.

### 6.2. The Paradox of Drug Accessibility Versus Viral Persistence in Gut Mucosa

Unlike the restricted penetration observed in lymph nodes, ART drug exposure in intestinal tissues is relatively abundant. Analysis by Asmuth et al. showed that antiviral drug concentrations in duodenal and rectal tissues from nearly all subjects exceeded the IC_50_ thresholds required for in vitro inhibition, indicating effective drug penetration into the intestinal mucosa [77]. However, a key paradox emerges: despite adequate drug concentrations, viral DNA and RNA remain detectable in intestinal tissues, and no correlation exists between drug levels and viral load [77]. This strongly suggests that the persistence of the gut viral reservoir cannot be simply attributed to insufficient drug penetration, but may instead be more closely related to local immune activation levels, cellular turnover rates, and the specialized gut-associated lymphoid tissue microenvironment. In other words, the gut represents a complex site that is “drug-accessible yet virus-refractory” [77].

### 6.3. The Dual Heterogeneity of the CNS as an Ultimate Sanctuary

Drug distribution within the CNS is characterized by dual heterogeneity: regional and cellular. At the macroscopic level, analysis of autopsy samples reveals substantial variability in the penetration of different antiviral drugs across 13 brain regions; for instance, DTG concentrations are lowest in the corpus callosum and highest in the choroid plexus [78]. At the microscopic level, this heterogeneity originates from the BBB itself, as all cellular components of the BBB (endothelial cells, pericytes, astrocytes) express functional drug-metabolizing enzymes and transporters [79]. Regional differences in the distribution and activity of these molecules lead to heterogeneous distribution of TFV, FTC, and their active metabolites within the brain [79]. Consequently, the CNS, as a whole, is not only subject to the global restriction of the BBB but, due to inter-regional metabolic differences, forms a “sanctuary within a sanctuary,” posing an unprecedented challenge to curative strategies.

## 7. Clearance Strategies Targeting the Three Major Reservoirs

Given the pronounced differences in drug penetration and microenvironment across the tissues discussed above, current HIV cure research is undergoing a profound shift from “universal” systemic interventions (e.g., latency reversal, transcriptional silencing, gene editing, stem cell transplantation, and immunotherapy) towards “tissue-specific” targeted strategies. Leveraging in-depth understanding of each tissue, researchers are designing innovative clearance strategies to precisely target the resilient viral reservoirs within lymph nodes, gut mucosa, and the CNS.

### 7.1. Breaching the Follicular Sanctuary in Lymph Nodes

Within lymph nodes, the virus primarily resides in germinal centers, which are poorly infiltrated by CD8^+^ T cells. Therefore, the core of therapeutic strategies lies in breaking this spatial segregation, either by enabling killer effector cells to enter follicles or by exposing reservoir cells within reach of cytotoxic cells.

Reprogramming Killer Effector Cell Homing: As previously mentioned, cytotoxic CD4^+^ T cells expressing granzyme A, while also constituting a reservoir, are found at higher levels in HIV elite controllers and are associated with viral control [80], suggesting these cells possess antiviral activity. This implies that inducing cytotoxic T follicular helper cells (CTfh) expressing CXCR5 (conferring follicular homing capacity) might represent a novel strategy to eliminate or suppress the lymph node viral reservoir. Anna Malyshkina’s team validated this concept in a Friend virus infection model, demonstrating that an agonistic anti-CD137 antibody could induce a cytotoxic program in Tfh, upregulating Eomes (a cytotoxicity-associated transcription factor) and granzyme B expression, generating CTfh with follicular homing capacity, concomitant with a reduction in the lymph node viral reservoir [81]. More critically, αCD137 combined with ART delayed viral rebound upon treatment interruption without disrupting lymph node architecture or interfering with humoral immune responses [81]. From the perspective of an HIV clinical cure, this strategy holds considerable promise, but its feasibility requires further validation in HIV/SIV infection models.

Engineering Targeted Cytotoxic Cells: This approach aims to: (a) equip killer cells with the ability to enter lymphoid follicles; (b) enable efficient recognition of Tfh, given their high PD-1 expression; and c) achieve precise killing of reservoir cells. Karsten Eichholz’s team constructed anti-PD-1 chimeric antigen receptor (CAR) T cells [82]. Animal experiments showed these cells could undergo long-term expansion in some individuals and deplete Tfh and other memory CD4^+^ T cells within lymph nodes, thereby clearing SIV-infected Tfh. However, off-target killing of PD-1^+^ CD8^+^ T cells by this approach raised significant safety concerns [82].

Enhancing and Inducing T Cell Immunity: As discussed earlier, follicular CD8^+^ T cells possess the capacity to infiltrate lymphoid follicles and germinal centers, playing a potential role in suppressing HIV/SIV reservoirs [32,33]. Studies have found that PD-1 inhibitors can promote their proliferation and enhance antiviral effects [83], but these agents carry risks of immune-related adverse events in clinical application, requiring further optimization and exploration of their safety and application strategies. Furthermore, advances in mRNA lipid nanoparticle technology have made it possible to induce potent and durable HIV-specific CD8^+^ T cell responses within lymphoid tissues [84]. Such “therapeutic vaccine” strategies hold promise for mobilizing a population of immune cells capable of residing in lymph nodes, synergizing with other strategies to clear the reservoir.

Harnessing Innate Immune Surveillance: NK cells, owing to their natural resistance to HIV infection, represent ideal “universal killers.” Preliminary studies of adoptive NK cell transfer combined with the IL-15 superagonist N-803 showed that donor-derived NK cells could persist in lymph nodes and rectal tissues, moderately reducing the frequency of viral RNA-positive cells [85]. Further mechanistic investigation revealed that this reduction correlated with N-803-mediated NK cell proliferation and NK group 2 member A expression on NK cells [86]. This provides proof-of-concept for utilizing innate immunity to target deep tissue reservoirs.

Disrupting the Physical Structure of the Sanctuary: An alternative strategy to reducing the reservoir involves depleting the B cell follicles that constitute the supportive microenvironment for Tfh. John K. Bui and colleagues designed and adoptively transferred CD20-targeting CAR-T cells into SHIV-infected animal models. These cells reversibly depleted B cell follicles. Although this did not directly reduce the viral reservoir [87], it offers insights for combination therapies: disrupting follicular structure followed by combining latency-reversing agents or effector cells might enable more complete clearance.

Summary: The core feature of the lymph node reservoir lies in the local microenvironment’s continuous “education” of immune cells under chronic inflammation, which not only fosters pro-latency subsets (e.g., CD73^+^CD4^+^ T cells) but also shapes subsets with clearance potential (e.g., CTfh). Consequently, CTfh-based strategies offer a unique pathophysiological advantage: they do not rely on exogenous killer cell infiltration into follicles but instead convert the very cells that HIV normally infects into effector cells that dismantle immune privilege from within. By contrast, while PD-1 blockade enhances T-cell function, the broad expression of PD-1 on various cells (Tfh, CD8^+^ T, etc.) carries safety risks of off-target elimination of antiviral immune cells or disruption of immune tolerance. Overall, a sequential combination of reversible follicular disruption (e.g., transient CD20 CAR-T) followed by CTfh adoptive transfer represents a promising future direction for targeted lymph node clearance.

### 7.2. Restoring the Immune Microenvironment of the Gut Mucosa

The challenge posed by the gut reservoir lies in its “drug-accessible yet virus-refractory” nature, suggesting that the core issue resides within local immunity. Therefore, the strategic focus shifts towards “ecological restoration.”

Targeting the Gut Homing Receptor α4β7: As discussed previously, the gut homing receptor α4β7 facilitates HIV infection. Beyond blocking its binding to the V2 loop of HIV/SIV gp120 and thereby inhibiting infection, the anti-α4β7 monoclonal antibody (vedolizumab) can also reshape the gut immune environment. Studies indicate that it operates through a triple mechanism: (a) at the structural level, the anti-α4β7 antibody mediates the reduction of lymphoid aggregates, which serve as viral foci [88]; (b) at the metabolic repair level, it restores the retinol metabolic pathway, re-establishing immune homeostasis (an effect dependent on the gut microbiota) [89]; (c) at the cellular phenotype level, it restores the normal mature phenotype of intestinal macrophages, which is lost during viral infection, and this restoration correlates with lower tissue viral loads [90]. It is worth noting that the efficacy of anti-α4β7 therapy is not firmly established [91]. In a non-human primate model, neither anti-α4β7 monoclonal antibody nor the dual antagonist (TR-14035) reduced plasma viral load or tissue viral reservoir size during SIV infection; indeed, TR-14035 administration during acute infection accelerated disease progression [91]. These findings suggest that simply blocking integrin homing pathways may be insufficient to control infection and could, under certain circumstances, have detrimental effects.

Nevertheless, from a strategic perspective, the gut differs from lymph nodes and the CNS in that ART penetrates this site relatively well; the persistence of virus stems primarily from chronic inflammation and barrier damage-driven “ecological imbalance” rather than from drug inaccessibility. Therefore, an “ecological restoration” strategy focused on repairing the microenvironment aims to re-establish local immune homeostasis and break the inflammation-virus persistence cycle. On this basis, combining such an approach with pharmacological inhibition or immunomodulation may enable effective control of the gut viral reservoir. Further investigation into the preventive or therapeutic application of integrin blockade is needed, and its combination with direct-acting antivirals warrants in-depth exploration.

### 7.3. Penetrating and Clearing the Cryptic Reservoir in the CNS

The dual heterogeneity of the CNS necessitates strategies that simultaneously address the challenges of “crossing the BBB” and “targeting specific cells.”

Precision Delivery Across the BBB: To achieve efficient brain entry, various nanotechnology platforms have been developed, primarily through active targeting, biomimetic delivery, and modulation of efflux pump function. For active targeting, CCR5 ligand-modified nanoparticles loaded with rilpivirine, combined with ultrasound and microbubbles to open the BBB, achieve precise anchoring to microglia and sustained release [92]; similarly, folic acid receptor-targeted lipid nanoparticles can selectively deliver drugs to activated macrophages [93]. In the realm of biomimetic strategies, biomimetic ionic liquid coatings enable nanoparticles to “hitchhike” on red blood cells, achieving efficient brain delivery and preferential uptake by microglia [94]. Beyond direct delivery, atomic-level studies have revealed that certain HIV protease inhibitors themselves can inhibit the efflux function of P-glycoprotein [95], offering a new pharmacological perspective for optimizing the CNS penetration of existing drugs.

Targeted Depletion of Myeloid Reservoirs: One approach involves direct depletion. The colony-stimulating factor 1 receptor inhibitor BLZ945 can effectively deplete perivascular macrophages in the brains of SIV-infected rhesus macaques and significantly reduce brain tissue viral DNA loads [96], demonstrating the feasibility of targeted depletion of CNS myeloid reservoirs in a non-human primate model. Another strategy is the “Block-and-Lock” approach. The traditional “Shock-and-Kill” strategy faces limitations including incomplete latency reversal and inflammatory risks. As an alternative, “Block-and-Lock” uses HIV transcription inhibitors (e.g., didehydro-Cortistatin A, dCA) to permanently lock the HIV promoter into a deep state of silencing. In vivo studies have shown that dCA markedly delays viral rebound and reduces rebound viral load after ART interruption [97], and dCA can cross the BBB and function within the CNS [98]. Additionally, some studies have attempted to add a clearance step building upon “Block-and-Lock”, termed “Block-Lock-and-Kill”. In this approach, viral transcription is first silenced using extracellular vesicles loaded with HIV Tat inhibitors; then, the transcription silencing-induced changes in membrane polarization are exploited to enable more efficient internalization of magnetoelectric cytotoxic nanoparticles [99]. This system has demonstrated feasibility in microglial cell models in vitro, but its in vivo safety, specificity, and added benefit over “Block-and-Lock” alone require further validation.

Indirect Microenvironmental Modulation: Beyond directly clearing infected cells, modulating the inflammatory microenvironment of the CNS can also weaken the “niche” of the viral reservoir. For instance, the honokiol hexafluoro analog can inhibit astrocyte activation, reduce the inflammatory phenotype of microglia, and reshape macrophage metabolism, shifting them from an activated towards a resting state, thereby indirectly reducing their potential as viral carriers [100].

Corticosteroids: Immunomodulation can potentially influence HIV reservoir dynamics during ART. A case study of an AIDS patient demonstrated that, during ART-mediated viral suppression, inflammation in the CNS caused by John Cunningham virus led to a significant increase in HIV viral load in the CSF and a modest increase in peripheral blood. Without altering the ART regimen, corticosteroid treatment (prednisone) significantly reduced the HIV viral load back to suppressive levels [64]. Such anti-inflammatory immunomodulation could potentially interrupt the inflammatory vicious cycle induced by CNS HIV, mitigating its damaging effects on brain tissue.

Summary: The relative compartmentalization of the CNS reservoir makes it the most tenacious viral sanctuary. The most realistic current strategy is to optimize drug delivery to maintain suppression, whereas further clearance strategies must be chosen with caution. The traditional “Shock-and-Kill” approach faces fundamental dilemmas in the CNS: (a) latency-reversing agents poorly cross the BBB; (b) even if virus is reactivated, the brain parenchyma lacks sufficient effector cells (e.g., CD8^+^ T cells) to execute clearance; and most critically, (c) viral reactivation itself may trigger neuroinflammation and exacerbate neuronal injury, counteracting the physiological goal of protecting vulnerable brain tissue. The CNS is characterized by tightly regulated immune responses that avoid collateral damage; any strong immune activation risks irreversible harm. In contrast, the “Block-and-Lock” strategy, which permanently locks proviruses into a deep state of silencing, is not only safer but also more feasible in the CNS. Candidate agents such as honokiol derivatives have shown potential to induce deep silencing. Furthermore, the “Block-Lock-and-Kill” strategy, which does not rely on immune-mediated clearance, represents an intriguing future direction, though its in vivo safety and specificity require further validation.

## 8. Conclusions and Outlook

This review aims to move beyond the traditional perspective of viewing the HIV reservoir as a single entity, systematically synthesizing and delving into the profound heterogeneity exhibited by proviruses within the three major anatomical sites: lymph nodes, gut mucosa, and the CNS. Although “long-term latency and refractoriness to clearance” represent a common outcome for reservoirs across tissues, the underlying biological logic driving this outcome—namely, the carrier roles, maintenance dynamics, and patterns of host–virus interplay—differs fundamentally. Clarifying these differences is an epistemological prerequisite for transitioning from a paradigm of “broad-spectrum attack” towards “precision modulation.”

First, considering the spatial structure and kinetic roles of the reservoirs, the three tissues constitute distinct nodes within the viral “persistence–dissemination” network. Lymph nodes function as both the central hub and the immune “training ground.” They are not only the largest reservoir but, due to their anatomical role as a “meeting hall” for immune cells, enable infected cells (particularly Tfh) to weave the reservoir into a dynamically interconnected network through systemic circulation. In viral rebound, lymph nodes play a pivotal role as the “epicenter.” More importantly, the immune microenvironment within lymph nodes (e.g., follicular structure, hypoxic conditions) does not passively harbor the virus; instead, under sustained “education” by chronic inflammation, it actively shapes a set of specialized cell populations with dual functions of “promoting latency” and “mediating antiviral activity” (e.g., CTfh, follicular CD8^+^ T cells). This reveals a profound insight: within the core “arsenal” of the immune system, a high-intensity “arms race” is underway between HIV and the host, with both sides “evolving.”

The gut mucosa represents the virus’s “ecological battlefield and inflammatory engine.” The persistence of this reservoir does not rely on physical isolation (drugs are accessible) but is rooted in its open ecology as the body’s largest barrier surface. What unfolds here is not an “arms race,” but an “ecological imbalance” driven by microbial translocation. The microenvironmental signaling network centered on RA and TGF-β “hijacks” normal immune tolerance and homing mechanisms into conduits for viral amplification. Meanwhile, the vicious cycle of barrier damage and chronic inflammation provides a continuous supply of “fuel” and a “breeding ground” for the reservoir. Thus, the gut reservoir is more akin to a “smoldering wildfire,” where the key lies in restoring the ecosystem rather than merely dousing with water.

The CNS functions as a “compartmentalized fortress and vicious cycle trap.” The persistence of its reservoir reaches an extreme, stemming from multiple “hard barriers”: physical (BBB), immune (antibody compartmentalization), and metabolic (drug efflux pumps). Unlike the networked nature of lymph nodes and the ecological nature of the gut, the CNS reservoir exhibits significant relative compartmentalization—infected cell clones primarily self-seed and evolve within this compartment. Its maintenance dynamic relies more heavily on a local inflammatory vicious cycle ignited by viral toxic proteins (Tat, Nef) and fueled by host pathological proteins (e.g., α-synuclein). This renders the CNS a “self-sustaining pathological microenvironment” actively co-constructed by the virus; though small in scale, it is the most resilient.

Independence and interconnectivity of tissue reservoirs: The viral reservoirs in lymph nodes, gut mucosa, and the CNS are not isolated compartments but rather form a hierarchical, dynamically interconnected network. The lymph node is the central hub of this network, yet each site operates in a “semi-autonomous” manner—capable of local self-maintenance while also being connected via the circulatory system, forming a decentralized distributed network. Regarding independence, reservoirs at all three sites are established early after infection and exhibit distinct decay kinetics under long-term ART, with each tissue microenvironment possessing autonomous maintenance capacity. Regarding interconnectivity, cross-tissue migration is a well-established systemic phenomenon: the most extensive and dynamic clonal exchange occurs between lymph nodes, peripheral blood, and the gut. Even in the relatively compartmentalized CNS, CSF contains infected T-cell clones that partially overlap with those in blood, and after treatment interruption, migratory CD4^+^ T cells are major contributors to CNS viral rebound. It is precisely this dual nature of independence and interconnectivity that necessitates distinct, tailored clearance strategies for different tissues, rather than a one-size-fits-all approach.

Second, based on these differences, targeted clearance strategies must also shift from “universal activation and killing” to “tissue-adapted modulation and clearance.” The differences in reservoir maintenance mechanisms across tissues dictate the logic for strategy selection, as defined by three principles. First, immune cell abundance determines the feasibility of “reactivation”. Lymph nodes are rich in effector cells (even if partially excluded), making “breaching spatial segregation to expose virus to immune firepower” a rational path; in contrast, the CNS is scarce in effector cells, where reactivation would only trigger harmful neuroinflammation, necessitating a shift to permanent silencing (Block-and-Lock). Second, drug accessibility determines the strategic focus. In the gut, drug penetration is adequate and viral persistence arises from microenvironmental imbalance rather than drug insufficiency, so the strategic focus should be “niche repair” (e.g., α4β7 blockade, anti-inflammation) rather than improved delivery. In the CNS, drug penetration is limited, making “precision delivery across barriers” a prerequisite for any strategy. Third, inflammation tolerance determines intervention intensity. Lymph nodes and the gut can tolerate a certain degree of immune activation, whereas the CNS is exquisitely sensitive to inflammation—this directly precludes the use of “Shock-and-Kill” in this site and favors silencing strategies that do not rely on immune activation. Clarifying these three principles is the first step toward a systematic, rational approach to intervention.

In summary, the in-depth dissection of HIV reservoir tissue heterogeneity is propelling cure research towards a new paradigm: the future breakthrough may not lie in discovering a single agent that eliminates all viruses, but rather in developing a systems modulation strategy based on a multi-dimensional understanding of reservoir dynamics. Such a strategy requires three interdependent lines of inquiry. First, we need to map a more complete tissue reservoir atlas. Current research must extend from the three core tissues (lymph nodes, gut, CNS) to other potential anatomical sites, including the spleen, lungs, and reproductive tract, to establish a comprehensive landscape of HIV persistence throughout the body. Second, efforts should focus on identifying the core regulatory interfaces between virus and host. Beyond studying viral structural genes, it is critical to investigate how accessory proteins (e.g., Tat, Nef, Vpr) hijack host epigenetic networks and immune-metabolic pathways. The goal is to identify cross-tissue, actionable nodes that are common to diverse cellular contexts. Third, we must develop multi-dimensional and synergistic intervention strategies. These should integrate complementary approaches—such as functional silencing via gene editing, precise modulation of immune checkpoints, and microenvironmental remodeling—to deliver tissue-specific interventions tailored to the distinct characteristics of each anatomical reservoir.

The ultimate objective is to transform replication-competent proviruses scattered across various tissues into harmless genomic fossils. This pursuit does not necessarily require a sterilizing cure in the classical sense. Rather, by deeply understanding the rules governing host–virus interplay across both temporal and spatial dimensions, we can achieve durable functional control of HIV within a dynamically balanced host system. Such control will rely on multi-site, multi-level, and multi-strategy synergistic interventions that progressively dismantle the survival niches HIV has established in different tissues. Only through this integrated approach can we pave a truly feasible path toward a clinical cure.

## Data Availability

No new data were created or analyzed in this study. Data sharing is not applicable to this article.

## Abbreviations

The following abbreviations are used in this manuscript:

ADCC	antibody-dependent cellular cytotoxicity
AIDS	acquired immunodeficiency syndrome
ART	antiretroviral therapy
BBB	blood–brain barrier
CAR	chimeric antigen receptor
CCR5	C-C chemokine receptor type 5
CNS	central nervous system
CSF	cerebrospinal fluid
CTCF	CCCTC-binding factor
CTfh	cytotoxic T follicular helper cells
CXCL13	C-X-C motif chemokine ligand 13
CXCR3	C-X-C chemokine receptor type 3
CXCR5	C-X-C motif chemokine receptor 5
dCA	didehydro-Cortistatin A
DCs	dendritic cells
DTG	dolutegravir
EREs	endogenous retroviral elements
FTC	emtricitabine
HIV	human immunodeficiency virus
IECs	intestinal epithelial cells
IL-7	interleukin 7
IL-12	interleukin 12
IL-15	interleukin 15
IPDA	intact proviral DNA assay
MAdCAM-1	mucosal addressin cell adhesion molecule-1
MHC II	major histocompatibility complex class II
mTOR	mammalian target of rapamycin
NK	natural killer
PBMCs	peripheral blood mononuclear cells
PCR	polymerase chain reaction
PD-1	programmed cell death protein 1
RA	retinoic acid
SHIV	simian-human immunodeficiency virus
SIV	simian immunodeficiency virus
TCR	T-cell receptor
Tem	effector memory CD4^+^ T cells
Tfc	follicular cytotoxic T cells
Tfh	follicular helper CD4^+^ T cells
TFV	tenofovir
TGF-β	transforming growth factor-β
TIGIT	T cell immunoreceptor with Ig and ITIM domains
TLR	Toll-like receptor
Tcm	central memory CD4^+^ T cells
Trm	tissue-resident memory CD4^+^ T cells
Ttm	transitional memory CD4^+^ T cells
